# Asymmetric Diels–Alder reaction with >C=P– functionality of the 2-phosphaindolizine-η^1^-P-aluminium(*O*-menthoxy) dichloride complex: experimental and theoretical results

**DOI:** 10.3762/bjoc.9.40

**Published:** 2013-02-18

**Authors:** Rajendra K Jangid, Nidhi Sogani, Neelima Gupta, Raj Kumar Bansal, Moritz von Hopffgarten, Gernot Frenking

**Affiliations:** 1 Department of Chemistry, University of Rajasthan, Jaipur 302004, India; 2 Department of Chemistry, IIS University, Jaipur 302020, India; 3 Fachbereich Chemie der Philipps Universität, D-35032 Marburg, Germany

**Keywords:** aluminium(*O*-menthoxy) dichloride, asymmetric synthesis, >C=P**–** functionality, DFT calculations Diels–Alder reaction

## Abstract

The Diels–Alder reaction of the 2-phosphaindolizine-η^1^-P-aluminium(*O*-menthoxy) dichloride complex with dimethylbutadiene was investigated experimentally and computationally. The >C=P**–** functionality of the complex reacts with 2,3-dimethylbutadiene with complete diastereoselectivity to afford [2 + 4] cycloadducts. Calculation of the model substrate, 3-methoxycarbonyl-1-methyl-2-phosphaindolizine-P-aluminium(*O*-menthoxy) dichloride (**7a**), at the DFT (B3LYP/6-31+G*) level reveals that the *O*-menthoxy moiety blocks the *Re* face of the >C=P**–** functionality, due to which the activation barrier of the Diels–Alder reaction of **7a** with 1,3-butadiene, involving its attack from the *Si* face, is lower. It is found that in this case, the exo approach of the diene is slightly preferred over the endo approach.

## Introduction

There is an increasing emphasis on the synthesis of optically pure compounds, as far as possible, for environmental, economic and social reasons. Using chiral auxiliaries for changing enantiotopic faces into diastereotopic faces is a common approach in asymmetric synthesis, which is one of the most attractive methods from the atom-economy point of view [1] for producing single enantiomers selectively. Over the past three decades, a variety of reactions allowing the formation of C**–**H, C**–**C, C**–**N, C**–**O and other bonds enantioselectively have been developed [2]. The chiral pool continues to be an attractive and economic source of enantiomerically pure chiral auxiliaries (ligands or modifiers) for enantioselective synthesis [3]. Two naturally occurring enantiomers of menthol and synthetically prepared (1*R*)-(+)-8-phenylmenthol have often been used as chiral auxiliaries [3–4].

Chiral phosphines constitute a very important group of ligands as their coordination compounds with transition metals have been extensively employed in asymmetric catalysis to convert achiral compounds into enantio-enriched products with high efficiency and enantioselectivity [5]. In many cases, chiral monophosphine ligands have been found to be more useful than chiral bisphosphines [6–8]. In view of this, efforts are always being made to obtain new chiral phosphines [9].

The first example of the Diels–Alder (DA) reaction with the >C=P**–** functionality of an azaphosphole was reported by Arbuzov and co-workers [10]. Subsequently, pioneering work by the research group of Appel established several interesting features associated with the DA reactions of phosphaalkenes [11–12]. Mathey and co-workers showed that 1*H*-phospholes underwent a 1,5-H shift followed by dimerization through a DA reaction [13]. The first DA reaction involving the –C=C–C=P– moiety of phosphinine as a diene was reported by Märkl and Lieb [14], while Mathey and Alcaraz showed that phosphinine could react as a dienophile as well, with the reaction taking place at the >C=P– functionality of phosphinine [15]. We recently compiled a review on the DA reactions involving the >C=P– functionality of various organophosphorus compounds wherein all these aspects have been discussed [16].

**Scheme 1:**
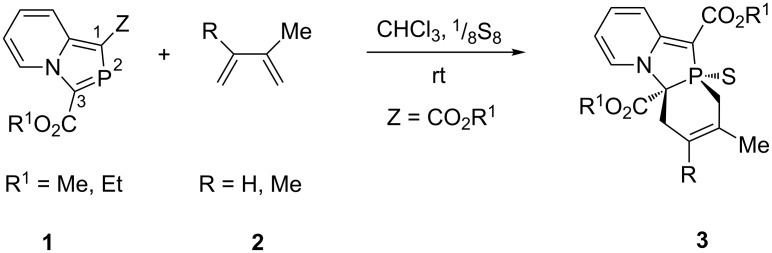
Diels–Alder reaction of 2-phosphaindolizines.

During the past few years, we have investigated the DA reaction with the >C=P**–** functionality of 1,3-azaphospholes theoretically as well as experimentally [16]. In this context, we found that 1,3-bis(alkoxycarbonyl)-2-phosphaindolizines (**1a**, Z = CO_2_R^1^) prepared through 1,5-electrocyclization of in situ generated bis(pyridinium ylidyl)phosphenium chlorides [17] lead to successful DA reaction [18–19], but 3-alkoxycarbonyl-2-phosphaindolizines having an electron-withdrawing group (EWG) only at the 3-position (**1b**, Z = Me) failed to undergo DA reaction even on heating under reflux in toluene alone or in the presence of sulfur [18] (Scheme 1).

It was demonstrated that the dienophilic reactivity of the >C=P– functionality of phosphinines could be enhanced by complexing the P-atom of phosphinine to a metal carbonyl, such as W(CO)_5_ [15,20]. Thus, the phosphinine-η^1^-P-W(CO)_5_ complex reacted with 1,3-dienes to afford [2 + 4] cycloadducts. By following the same strategy, we recently reported our theoretical and experimental results of the DA reactions of 2-phosphaindolizine-η^1^-P-AlEtCl_2_ complexes [21]. Theoretical calculations at the DFT (B3LYP/6-31+G**) level indicated lowering of the activation barrier by 6 kcal mol^–1^ for the reaction of σ^2^,λ^3^-*P*-coordinated 2-phosphindolizine to methylaluminium dichloride with 1,3-butadiene as compared to that for the corresponding reaction of the uncomplexed 2-phosphaindolizine. The cycloadducts so obtained were well characterized by ^1^H, ^31^P and ^27^Al NMR data and, thus, confirmed the theoretical results.

Koga and co-workers [22] used, for the first time, chiral (−)-menthoxyaluminium dichloride, derived from the reaction of (–)-menthol with ethylaluminium dichloride, for the asymmetric catalytic DA reaction of methacrolein with cyclopentadiene leading to 66% ee. It led to the development of a variety of chiral aluminium and other organometallic catalysts for use in organic synthesis [23–25]. In view of this, it was considered interesting to prepare a 2-phosphindolizine-η^1^-P complex by using a chiral Lewis acid, (−)-menthoxyaluminium dichloride, and to investigate experimentally and theoretically the diastereoselectivity of its DA reaction. The results are described herein.

## Results and Discussion

### Experimental results

**Scheme 2:**
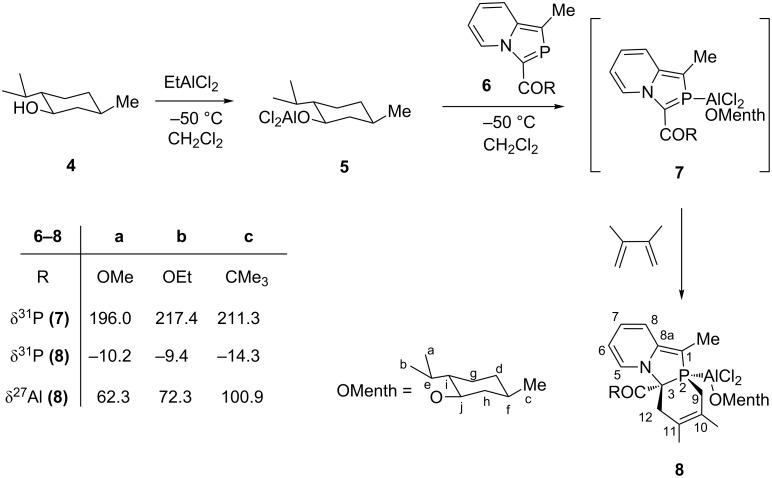
Diels–Alder reaction of 2-phosphaindolizine-η^1^-P-aluminium(*O*-menthoxy) dichloride with 2,3-dimethylbutadiene.

(2-Phosphaindolizine-η^1^-P)-Al(*O*-menthoxy)Cl_2_ (**7**) was generated in situ by reacting 2-phosphaindolizine with (*O*-menthoxy)aluminium dichloride (**5**); formation of the complex is confirmed by ^31^P NMR (δ 196.0–217.4 ppm). Coordination of the σ^2^,λ^3^-P atom of 2-phosphaindolizine to (*O*-menthoxy)aluminium dichloride causes a downfield shift in the ^31^P NMR signal by δ 34–55 ppm, which is in accordance with the previous results [26–27]. An attempt to isolate the complex was, however, unsuccessful. 2,3-Dimethylbutadiene was then added and the progress of the reaction was monitored by ^31^P NMR. The reaction proceeded with complete diastereoselectivity, and in each case, only one isomer (**8**) was formed, as shown by the ^31^P NMR of the reaction mixture (Scheme 2).

The cycloadducts **8** are pale yellow, fine, crystalline solids, sparingly soluble in methylene chloride and chloroform. Their structures have been confirmed on the basis of ^31^P, ^27^Al, ^1^H and ^13^C NMR studies. The upfield ^31^P NMR chemical shifts in the range of δ –9.4 to –14.3 ppm (Scheme 2) are in conformity with those reported for the cycloadducts resulting from the DA reactions of P-W(CO)_5_ complexes of λ^3^-phosphinines [15]. The ^27^Al NMR signal at δ 62.3 to 100.9 ppm (Scheme 2) indicates fourfold coordination of the aluminium atom [28]. In addition, a broad signal at δ 44.9 to 51.1 ppm (∆ν_1/2_ 5707 to 8834 Hz) and the absence of ^31^P–^27^Al coupling may be due to exchange of the ligand [29]. ^13^C NMR studies have been used extensively in the characterization of azaphospholes and their [2 + 4] cycloadducts due to their characteristic ^13^C–^31^P coupling constants [30–32]. In view of this, the ^13^C NMR spectrum of a representative product **8a** was recorded. The signals of the carbon atoms directly bonded to the phosphorus atom, namely C1 (δ = 132.7 ppm, ^1^*J*_PC_ = 36.0 Hz), C3 (δ = 54.5 ppm, ^1^*J*_PC_ = 31.7 Hz) and C9 (δ = 33.6 ppm, ^1^*J*_PC_ = 44.5 Hz) are identified readily by large values of ^1^*J*_PC_ [33–34]. The ^13^C NMR signals due to the *O*-menthoxy moiety were assigned on the basis of the reported results [35].

### Mode of action of the catalyst

**Scheme 3:**
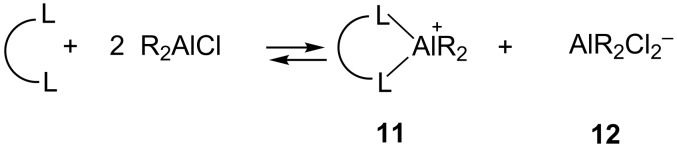
Formation of the cationic 1:1 complex of the dienophile and dialkylaluminium.

In the DA reactions catalysed by excess dialkylaluminium chloride, formation of the chelate complex cation **11** of the dienophile (Scheme 3) has been established experimentally [36–38], and the high reactivity of the dienophile in the presence of the organoaluminium catalyst was attributed to the formation of this cationic species.

**Scheme 4:**
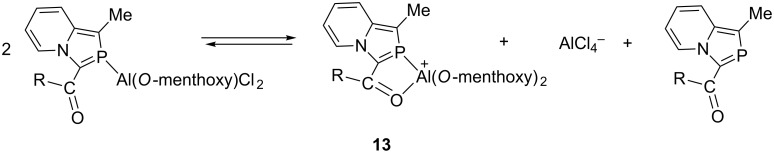
Disproportionation of the 1:1 complex of 2-phosphaindolizine and Al(*O*-menthoxy)Cl_2_.

Later, Tietze et al. [39] rationalized higher reactivity and observed stereoselectivity resulting from the formation of the cationic complex on the basis of computational calculations. Recently, Yu and co-workers [40] investigated theoretically and experimentally the InCl_3_-catalyzed cycloisomerisation of 1,6-enynes and demonstrated InCl_2_^+^ to be the actual catalytic species participating in the reaction. In this context, it has been emphasized that identifying the real catalytic species may be very challenging, because in many cases impurities in the catalysts act as the real catalytic species [41]. As one of the referees pointed out this possibility, we carefully checked for the formation of a chelate cationic complex **13** on addition of the catalyst. After adding 2-phosphaindolizine (1 equiv) solution to the previously generated (*O*-menthoxy)aluminium dichloride solution, ^31^P NMR of the resulting solution was performed, in which only one signal in the range of δ 196–211 ppm corresponding to the (2-phosphaindolizine-η^1^-P)-Al(*O*-menthoxy)Cl_2_ complex was observed, and no ^31^P NMR signal for the uncomplexed 2-phosphaindolizine was detected, thus ruling out formation of the cationic species **13** (Scheme 4).

Furthermore, it has been established by X-ray crystal structure studies that Cr(CO)_5_ is coordinated to the phosphorus atom only, and no chelate complex involving the σ^2^,λ^3^-P atom and carbonyl oxygen atom is formed [27]. As reported recently, the DFT calculations reveal that the activation energy of the DA reaction is lowered only if the aluminium catalyst is coordinated to the phosphorus atom; when it is coordinated to the carbonyl oxygen atom, the activation energy barrier is rather high as compared to that for the DA reaction of the uncomplexed 2-phosphaindolizine [42]. Computational calculations also show that the conformation of 2-phosphaindolizine corresponding to the global minimum has phosphorous and carbonyl oxygen atoms in the antiperiplanar positions [35], thus reducing the possibility of chelate formation.

### Theoretical results

**Scheme 5:**
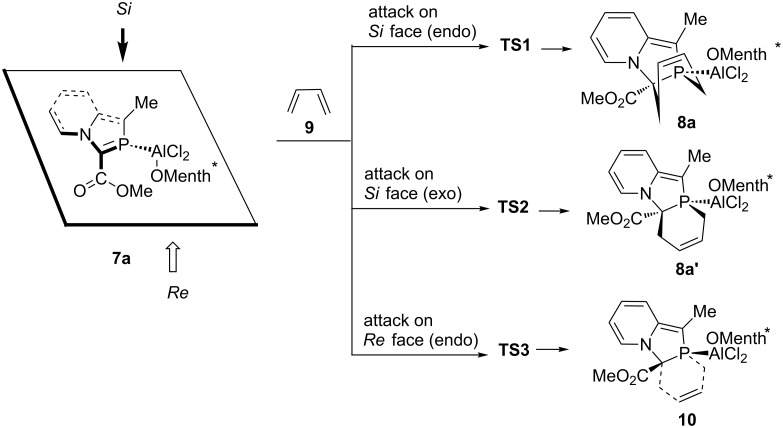
Attack of 1,3-butadiene on *Si* and *Re* faces of >C=P– functionality of 2-phosphaindolizine complex.

We then investigated theoretically the mode of action of the chiral auxiliary in directing the complete diastereoselectivity of the DA reactions. The following model DA reactions (Scheme 5) were calculated at the DFT (B3LYP/6-31+G*) level.

### Computational calculations

It has been reported that for determining activation free energies and enthalpies of the pericyclic reactions, computational calculations at the B3LYP/6-31+G(d) level are very suitable [43–45]. Furthermore, the X-ray crystal investigation in one case confirmed the endo-structure of the resulting [4 + 2] cycloadduct [19]. In view of this, we also carried out computational calculations using the hybrid functional of Becke [46] and Lee, Yang and Parr [47]. Geometry optimizations of the reactants, the transition states and the cycloadducts were performed at the B3LYP/6-31+G* level. Stationary points were analysed by frequency calculations at the same level to confirm their character as local minima or transition structures. IRC calculations were performed in order to validate the connection of each transition state with the respective reactants and products. The solvent effect was computed by carrying out the single-point energy calculations of the gas-phase optimized geometries using the polarized continuum model (PCM). The Gaussian 03 program package [48] was used for all calculations.

#### Optimized geometries

**Figure 1:**
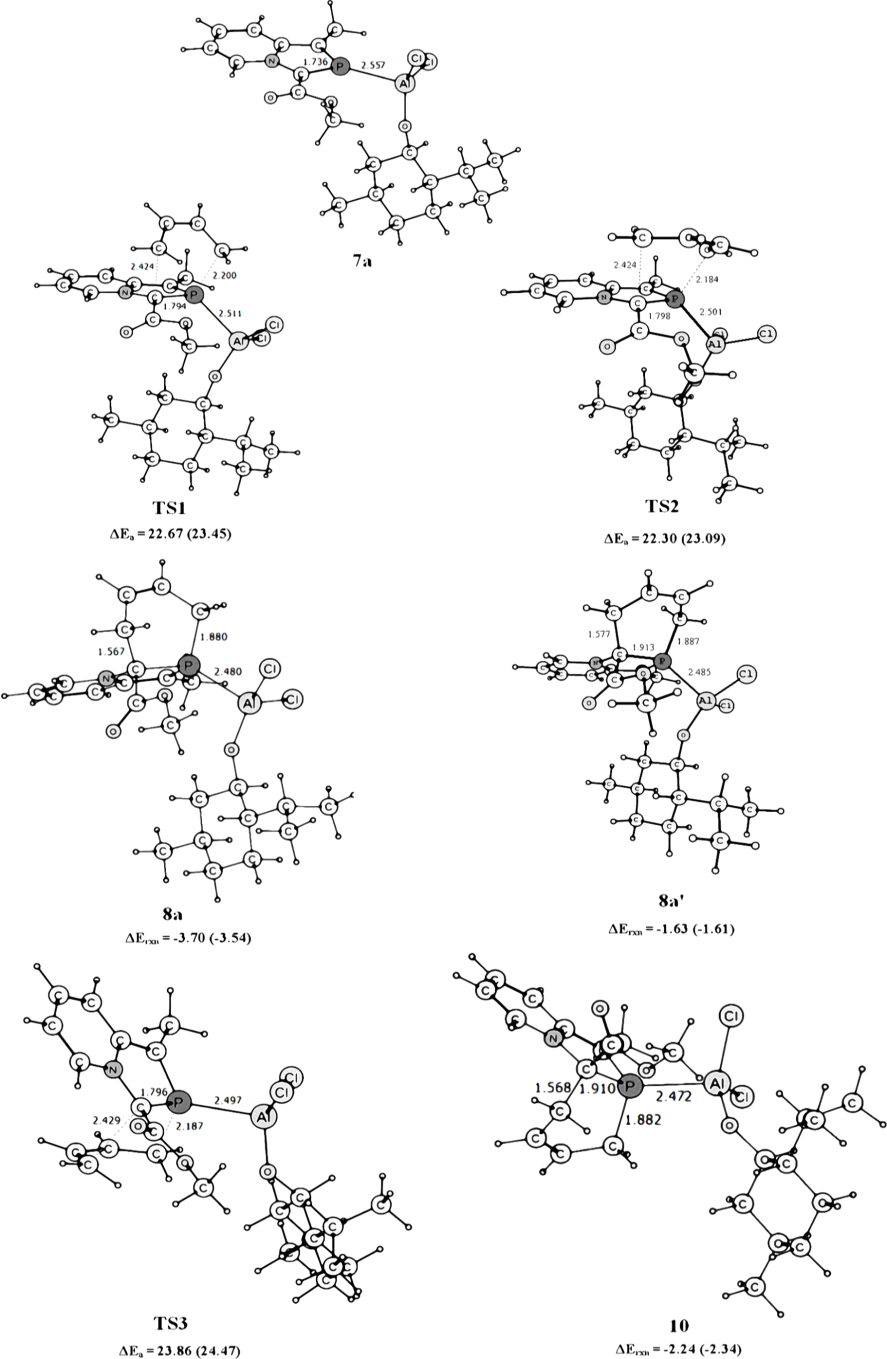
Geometries of 2-phosphaindolizine-η^1^-P-aluminium(*O*-menthoxy) dichloride, the transition structures, and the products optimized at the B3LYP/6-31+G* level in the gas-phase. The relative activation and reaction energies obtained in methylene chloride are given in parentheses.

Optimized geometries of (2-phosphaindolizine-η^1^-P)-Al(*O*-menth*)Cl_2_ (**7a**), the transition structures (**TS1**, **TS2** and **TS3** ), and the products (**8a, 8a’** and **10**) are shown in Figure 1.

The optimized geometry of **7a** (Figure 1) reveals that the menthol moiety shields the *Re* face of the >C=P– functionality in the coordinated 2-phosphaindolizine molecule. Attack of the 1,3-butadiene molecule from the less hindered *Si* face leads to the transition structures **TS1** (endo) and **TS2** (exo) and the products **8a** and **8a’**, respectively. On the other hand, attack of the diene from the sterically hindered *Re* face leads to the transition structure **TS3** and the product **10**.

#### Energetics

Ab initio investigations of the DA reaction of phosphaethene with 2*H*-phosphole [49] and with 1,3-butadiene [50–52] revealed low activation energies and a preference for the endo approach. In the present case, endo attack of the 1,3-butadiene molecule from the sterically more hindered *Re* face expectedly involves the higher-energy transition structure **TS3**. As regards the attack of the diene molecule from the sterically less hindered *Si* face, reactions involving both endo and exo approaches have been computed, and in contrast to the previous results, we find that the activation energy barrier for the exo approach involving **TS2** is smaller than for the endo approach via **TS1**, by ca. 0.3 kcal mol^−1^. Presence of the bulky O-menthoxy moiety possibly makes the exo approach more preferable as compared to the endo approach. In methylene chloride, activation-energy barriers are increased by ca. 1 kcal mol^−1^. All the reactions are moderately exothermic, and exothermicity remains almost unaffected in methylene chloride.

#### Kinetics of the reactions

Standard-state entropies and entropy changes of different species, as well as the enthalpies and the Gibbs free energies of the computed reactions (as shown in Scheme 5) are given in Table 1.

**Table 1:** Standard state entropies *S*^0^, entropy change Δ*S*, reaction enthalpies Δ*H*^0^ and reaction Gibbs free energies Δ*G*^0^.

Entry	Species	*S*^0^ (cal K^−1^ mol^−1^)	Δ*S* (cal K^−1^ mol^−1^)^a^	Δ*H*^0^ (kcal mol^−1^ )	Δ*G*^0^ (kcal mol^−1^ )

1	**7a**	211.1	–		
2	**9**	66.1	–		
3	**TS1**	231.4	−45.8		
4	**TS2**	227.4	−49.8		
5	**TS3**	231.9	−45.3		
6	**8a**	225.7	−51.5	−3.70	+11.65
7	**8a’**	225.9	−51.3	−1.63	+13.67
8	**10**	225.8	−51.4	−2.24	+13.08

^a^The relative entropy change; Δ*S* values have been obtained by subtracting the sum of the *S*^0^ values of **7a** and **9** from the *S*^0^ value of the respective transition structure or the product.

The entropy effects have been found to play a major role in enzyme catalysis [53]. However, in the present case, the entropy difference between the **TS1** and **TS3** is negligible and does not appear to play significant role. On the other hand, entropy effects favour the endo approach over the exo approach from the *Si* face. Although the three reactions are endergonic, the reaction involving the endo approach of the diene from the less hindered *Si* face is preferred.

Π-Facial selectivity in the DA reactions has been investigated theoretically and the results have been found to be consistent with the experimentally observed results [54]. Origin of the diastereoselectivity observed in the cycloisomerisations of triynes has been correlated with the Gibbs free energies of the diastereomers calculated at the DFT B3LYP/TZV+P level; a difference of ca. 2 kcal mol^−1^ of Gibbs free energy corresponded to 84% diastereoselectivity [55]. In the present case also, the observed diastereoselectivity originates from the *Re* face being effectively blocked by the *O*-menthoxy moiety, thus making the diene attack the >C=P– functionality from the side of the *Si* face. In this case, the difference between the Gibbs free energies of **8a** and **8a’** is found to be 2.02 kcal mol^−1^ in favour of the former.

The results suggest that the proposed mechanism involving the preferred attack of the diene from the *Si* face leading to the observed diastereoselectivity is valid, but the calculated absolute values for the energy barrier from this method are possibly too high.

## Conclusion

The >C=P– functionality in 2-phosphaindolizines can be activated by coordinating the phosphorus atom to the Al(*O*-menthoxy)Cl_2_ moiety when it reacts with 2,3-dimethylbutadiene with complete diastereoselectivity. Computational calculations of the model DA reactions of (3-methoxycarbonyl-1-methyl-2-phosphaindolizine-η^1^-P)-Al(*O*-menth*)Cl_2_ with 1,3-butadiene reveal that the *Re* face is sterically hindered, and consequently, attack of the diene occurs preferentially from the *Si* face. Thermochemical data also support a preferential endo attack of the diene from the *Si* face. However, the absolute values for the energy barrier calculated for this method are possibly too high.

## Experimental

### Materials

Chemicals and solvents were purchased from Sigma-Aldrich. Solvents were dried according to the reported procedures. All the reactions were carried out in oxygen-free dry nitrogen under perfectly anhydrous conditions by using the Schlenk technique. 2-Phosphaindolizines (Scheme 2) were prepared by the [4 + 1] cyclocondensation method from the reaction of the respective 1-alkyl-2-ethylpyridinium bromide with phosphorus trichloride in the presence of triethylamine, as described in literature [56].

### Analysis and characterisation of the products

Melting points were determined on a Tempo apparatus and are uncorrected. NMR spectra were recorded on a Jeol EX-300 MHz spectrometer: ^31^P NMR at a frequency of 121.50 MHz (using H_3_PO_4_ as the external reference), ^1^H NMR at a frequency of 300.40 MHz and ^13^C NMR at a frequency of 75.50 MHz (using TMS as the internal reference), and ^27^Al NMR at a frequency of 78.17 MHz (using Al(OiPr)_3_ as the external reference).

### General method

A solution of (−)-menthoxyaluminium dichloride (**5**) (Scheme 2) was generated in situ [24] by adding ethylaluminium dichloride (4.6 mmol, 2.5 mL of 1 M solution in toluene) to a solution of (−)-menthol (4.6 mmol) (**4**) in CH_2_Cl_2_ under constant stirring at room temperature. This was followed by the addition of a solution of 2-phosphindolizine **6** (4.6 mmol) in CH_2_Cl_2_ (20mL) upon which an intense yellow colour developed (Scheme 2). After stirring for 30 minutes, the reaction mixture was cooled to −50 °C and a fivefold excess of 2,3-dimethylbutadiene (23 mmol, 1.8 g, 2.5 mL) was added under continuous stirring. The solution was then allowed to warm up to room temperature. After stirring of the reaction mixture overnight, completion of the reaction was revealed by the presence of only one signal (δ −9.4 to −14.3 ppm) in the ^31^P NMR spectrum. The solution was concentrated under vacuum to about 1/3 of its volume and left in a refrigerator after the addition of a few drops of hexane. Fine pale yellow crystals of the cycloadduct **8** deposited were separated, washed with hexane, and dried under vacuum.

**Compound 8a:** Yield 50%; mp 174–176 °C; ^31^P NMR δ −10.2; ^1^H NMR (300 MHz, CDCl_3_, TMS) δ 9.79 (d, ^3^*J*_HH_ = 7.3 Hz, 1H, 5-H), 7.41 (d, ^3^*J*_HH_ = 9.0 Hz, 1H, 8-H), 7.06 (dd, ^3^*J*_HH_ = 9.0, 6.6 Hz, 1H, 7-H), 6.81 (dd, ^3^*J*_HH_ = 7.3, 6.6 Hz, 1H, 6-H), 3.83 (s, 3H, -OMe), 3.34 (td, ^3^*J*_HH_ = 9.0, 4.2 Hz, 1H, *j*-H), 2.53 (d, ^3^*J*_PH_ = 12.0 Hz, 3H, 1-Me), 2.10 (m, 1H, *e*-H), 1.90 (m, 1H, *h*-H), 1.64–1.49 (unresolved m, 6H, 9-CH_2_, 12-CH_2_, *d*-H, *g*-H), 1.41–1.25 (unresolved m, 7H, 10-Me, 11-Me, *f*-H), 1.04 (m, 1H, *i*-H), 0.96–0.69 (m, 3H, *d'*-H, *g'*-H, *h'*-H), 0.84 (d, ^3^*J*_HH_ = 9.0 Hz, 3H, *b*-CH_3_), 0.82 (d, ^3^*J*_HH_ = 10.8 Hz, 1H, *c*-H), 0.71 (d, ^3^*J*_HH_ = 7.2 Hz, 3H, *a*-CH_3_); ^13^C NMR (75.5 MHz, CDCl_3_ + DMSO-*d*_6_, TMS) δ 169.5 (C8a), 141.1 (d, ^2^*J*_PC_ = 38.5 Hz, CO), 133.7 (d, ^3^*J*_PC_ = 15.0 Hz, C5), 132.7 (d, ^1^*J*_PC_ = 36.0 Hz, C1), 130.5 (d, ^3^*J*_PC_ = 19.0 Hz, C8), 126.9 (d, ^2^*J*_PC_ = 3.0 Hz, C10), 121.6 (d, ^3^*J*_PC_ = 6.0 Hz, C11), 58.8 (OMe), 54.5 (d, ^1^*J*_PC_ = 31.7 Hz, C3), 36.4 (11-CH_3_), 33.6 (d, ^1^*J*_PC_ = 44.5 Hz, C9), 30.3 (d, ^3^*J*_PC_ = 2.3 Hz, 10-CH_3_), 26.1 (d, ^2^*J*_PC_ = 6.0 Hz, C12), 17.9 (d, ^2^*J*_PC_ = 19.9 Hz, 1-Me); ^13^C NMR signals of *O*-menthoxy moiety: δ 75.1 (j-C), 50.1 (i-C), 39.4 ((h-C), 32.8 (d-C), 27.8 (f-C), 27.2 (e-C), 25.4 (g-C), 22.9 (c-C), 20.6 (a,b-C); anal. calcd for C_26_H_39_NO_3_Cl_2_PAl: C 57.57%, H 7.25%, N 2.58%; found: C 57.42%, H 7.34%, N 2.51%.

**Compound 8b:** Yield 46%; mp 182–184 °C; ^31^P NMR δ −9.4; ^1^H NMR (300 MHz, CDCl_3_, TMS) δ 9.85 (d, ^3^*J*_HH_ = 7.5 Hz, 1H, 5-H), 7.46 (d, ^3^*J*_HH_ = 9.0 Hz, 1H, 8-H), 7.25 (dd, ^3^*J*_HH_ = 9.0 Hz, 1H, 7.4, 7-H), 6.88 (t, ^3^*J*_HH_ = 7.4 Hz, 1H, 6-H), 4.38 (q, ^3^*J*_HH_ = 7.2 Hz, 2H, -OCH_2_), 3.45 (m, 1H, *j*-H), 2.59 (d, ^3^*J*_PH_ = 12.0 Hz, 3H, 1-Me), 2.15 (m, 1H, *e*-H), 1.95 (m, 1H, *h*-H), 1.73–1.55 (unresolved m, 6H, 9-CH_2_, 12-CH_2_, *d*-H, *g*-H), 1.43–1.35 (multiplet, 7H, 10-CH_3_, 11-CH_3_, *f*-H), 1.39 (t, ^3^*J*_HH_ = 7.2 Hz, 3H, -OCH_2_*CH_3_*), 1.14 (m, 1H, *i*-H), 0.99–0.83 (unresolved m, 9H, *d'*-H, *g'*-H, *h'*-H, *b*-CH_3_, *c*-CH_3_), 0.80 (d, ^3^*J*_HH_ = 6.9 Hz, 1H, *a*-CH_3_); anal. calcd for C_27_H_41_NO_3_Cl_2_PAl: C 58.28%, H 7.43%, N 2.52%; found: C 57.96%, H 7.59%, N 2.47%.

**Compound 8c:** Yield 49.8%; mp 169–171 °C; ^31^P NMR δ −14.3; ^1^H NMR (300 MHz, CDCl_3_, TMS) δ 10.25 (d, ^3^*J*_HH_ = 7.5 Hz, 1H, 5-H), 7.48 (d, ^3^*J*_HH_ = 7.8 Hz, 1H, 8-H), 7.24 (t, ^3^*J*_HH_ = 7.5 Hz, 1H, 7-H), 6.88 (t, ^3^*J*_HH_ = 7.2 Hz, 1H, 6-H), 3.41 (m, 1H, *j*-H), 2.61 (d, ^3^*J*_PH_ = 12.3 Hz, 3H, 1-Me), 2.15 (m, 1H, *e*-H), 1.92 (m, 1H, *h*-H), 1.73–1.43 (multiplet, 6H, 9-CH_2_, 12-CH_2_, *d*-H, *g*-H), 1.56 (s, 9H, -CMe_3_), 1.30 (s, 3H, 10-Me), 1.25 (s, 3H, 11-Me), 1.15 (m, 1H, *i*-H), 1.02–0.72 (unresolved m, 9H, *d'*-H, *g'*-H, *h'*-H, *b*-CH_3_, *c*-CH_3_), 0.80 (d, ^3^*J*_HH_ = 7.2 Hz, 3H, *a*-CH3); anal. calcd for C_29_H_45_NO_2_Cl_2_PAl: C 58.10%, H 7.57%, N 2.34%; found: C 57.92%, H 7.65%, N 2.28%.

## Supporting Information

File 1 Cartesian coordinates of the geometries optimized (Table S1) and total energies of reactants, transition structures and products in the gas phase and in methylene chloride (Table S2) at the B3LYP/6-31+G* level.
