# Supplementary material for: Asymmetric Diels–Alder reaction with >C=P– functionality of the 2-phosphaindolizine-η1-P-aluminium(O-menthoxy) dichloride complex: experimental and theoretical results
Source: Beilstein J Org Chem. 2013 Feb 18;9:392–400. doi: 10.3762/bjoc.9.40 (PMC3596042; doi:10.3762/bjoc.9.40)
Supplement: File 1 — Cartesian coordinates of the geometries optimized (Table S1) and total energies of reactants, transition structures and products in the gas phase and in methylene chloride (Table S2) at the B3LYP/6-31+G* level. [file Beilstein_J_Org_Chem-09-392-s001.pdf]

Supporting Information

for

Asymmetric Diels–Alder reaction with >C=P– functionality of the 2-phosphaindolizine-η^1-P-aluminium(O-menthoxy) dichloride complex: experimental and theoretical results

Rajendra K. Jangid[1], Nidhi Sogani[2], Neelima Gupta[1], Raj K. Bansal[2]*, Moritz von Hopffgarten[3] and Gernot Frenking[3]*

Address: [1]Department of Chemistry, University of Rajasthan, Jaipur 302004, India, [2]Department of Chemistry, IIS University, Jaipur 302020, India and [3]Fachbereich Chemie der Philipps Universität, D-35032 Marburg, Germany

Email: Raj K. Bansal* - bansal56@gmail.com; Gernot Frenking* - frenking@chemie.uni-marburg.de

* Corresponding author

Cartesian coordinates of the geometries optimized (Table S1) and total energies of reactants, transition structures and products in the gas phase and in methylene chloride (Table S2) at the B3LYP/6-31+G* level.

Table S1: Cartesian coordinates of the geometries optimized at the B3LYP/6-31+g* level.

7a

C	6.122176000	1.316326000	-0.858090000
C	5.947601000	1.431747000	0.546560000
C	4.803422000	0.987149000	1.147044000
N	3.792853000	0.415655000	0.390484000
C	3.929939000	0.284985000	-1.006005000
C	5.124908000	0.750907000	-1.612779000
C	2.587736000	-0.073449000	0.870639000
C	2.211283000	-0.054946000	2.284567000
O	2.883552000	0.378869000	3.211340000
C	2.820314000	-0.312323000	-1.633807000
C	2.738942000	-0.560763000	-3.115894000
P	1.632351000	-0.698925000	-0.437137000
Al	-0.719934000	-1.699670000	-0.485162000
O	-1.750207000	-0.504426000	0.172474000
O	0.990673000	-0.601283000	2.435106000
C	0.466171000	-0.681148000	3.773820000
Cl	-0.488115000	-3.459160000	0.690477000
Cl	-0.845332000	-2.018837000	-2.607220000
H	6.718039000	1.874003000	1.169205000
H	4.604145000	1.042083000	2.207838000
H	7.034926000	1.671845000	-1.325977000
H	5.222205000	0.645164000	-2.687819000
H	1.786216000	-1.020899000	-3.387475000
H	2.831155000	0.375413000	-3.681932000
H	3.540498000	-1.232273000	-3.450325000
H	-0.511587000	-1.148346000	3.663475000
H	1.122090000	-1.292319000	4.399083000
H	0.375739000	0.320040000	4.202970000
C	-2.510553000	0.571198000	-0.364385000
C	-1.687410000	1.867101000	-0.301969000
H	-2.742046000	0.361354000	-1.421140000
C	-2.456804000	3.098276000	-0.809974000
H	-1.379717000	2.027478000	0.742517000
H	-0.765545000	1.733340000	-0.885758000
C	-3.785840000	3.229832000	-0.048029000
C	-1.608266000	4.372875000	-0.714850000
H	-2.697814000	2.931443000	-1.872887000
C	-4.614068000	1.937741000	-0.116437000
H	-3.571850000	3.472909000	1.004890000
H	-4.367302000	4.071061000	-0.450858000
C	-3.837759000	0.714455000	0.414324000
H	-5.543567000	2.067497000	0.451222000
H	-4.912152000	1.761288000	-1.160408000
H	-3.550162000	0.927751000	1.457401000
C	-4.666552000	-0.598805000	0.450846000
H	-2.154193000	5.244095000	-1.098758000
H	-1.333288000	4.584411000	0.327395000
H	-0.679406000	4.278722000	-1.292055000
H	-3.967795000	-1.379524000	0.776199000
C	-5.789634000	-0.537029000	1.500642000
C	-5.235971000	-1.025190000	-0.914241000
H	-5.679208000	-2.026290000	-0.843264000
H	-6.025367000	-0.343460000	-1.256638000
H	-4.466290000	-1.065782000	-1.693515000
H	-6.285455000	-1.511675000	1.593180000
H	-5.397617000	-0.268349000	2.490277000
H	-6.562103000	0.196056000	1.235309000

9

C	-0.326701000	0.652114000	0.000000000
C	0.326701000	1.826242000	0.000000000
C	0.326701000	-0.652114000	0.000000000
C	-0.326701000	-1.826242000	0.000000000
H	-0.202963000	2.774563000	0.000000000
H	1.414155000	1.872857000	0.000000000
H	-1.417538000	0.646904000	0.000000000
H	1.417538000	-0.646904000	0.000000000
H	0.202963000	-2.774563000	0.000000000
H	-1.414155000	-1.872857000	0.000000000

TS1

C	4.069116000	3.448220000	-0.508837000
C	3.848461000	3.302569000	0.893590000
C	3.169909000	2.215223000	1.362428000
N	2.681258000	1.258592000	0.497397000
C	2.874216000	1.360504000	-0.899780000
C	3.598351000	2.501227000	-1.375525000
C	2.053738000	0.063310000	0.889890000
C	1.248487000	0.001104000	2.120974000
O	1.154647000	0.871195000	2.972606000
C	2.305058000	0.341271000	-1.651161000
C	2.398463000	0.230483000	-3.149219000
P	1.607183000	-0.864329000	-0.579786000
Al	-0.756520000	-1.678227000	-0.813724000
O	-1.737042000	-0.543752000	0.020634000
O	0.630506000	-1.196882000	2.200121000
C	-0.289107000	-1.385583000	3.295340000
Cl	-0.681012000	-3.639003000	0.059632000
Cl	-0.943049000	-1.723244000	-2.960815000
H	4.208583000	4.043167000	1.598818000
H	2.947785000	2.048693000	2.406586000
H	4.615943000	4.308707000	-0.883759000
H	3.761716000	2.587361000	-2.444045000
H	1.634849000	-0.446159000	-3.543454000
H	2.245837000	1.202481000	-3.634239000
H	3.379518000	-0.149274000	-3.474590000
H	-0.677067000	-2.394810000	3.163285000
H	0.234356000	-1.283396000	4.249408000
H	-1.096160000	-0.653113000	3.229461000
C	-2.582926000	0.524834000	-0.391286000
C	-1.797038000	1.845171000	-0.360434000
H	-2.916501000	0.347304000	-1.426529000
C	-2.652827000	3.069370000	-0.728210000
H	-1.383657000	1.975404000	0.651340000
H	-0.941507000	1.765930000	-1.045556000
C	-3.893202000	3.125963000	0.178075000
C	-1.836079000	4.366775000	-0.670100000
H	-3.004180000	2.937513000	-1.764997000
C	-4.687339000	1.811652000	0.138359000
H	-3.572794000	3.331163000	1.211883000
H	-4.538526000	3.963935000	-0.121183000
C	-3.824861000	0.591641000	0.525267000
H	-5.553568000	1.884552000	0.807638000
H	-5.090771000	1.672939000	-0.875141000
H	-3.436139000	0.768801000	1.542179000
C	-4.609196000	-0.748259000	0.584815000
H	-2.444375000	5.233072000	-0.960372000

H	-1.459659000	4.548074000	0.345692000
H	-0.970414000	4.324178000	-1.343401000
H	-3.856798000	-1.523492000	0.776893000
C	-5.598888000	-0.778907000	1.762796000
C	-5.327925000	-1.117774000	-0.725127000
H	-5.759129000	-2.123795000	-0.649543000
H	-6.150906000	-0.426597000	-0.947393000
H	-4.648828000	-1.118869000	-1.585630000
H	-6.047402000	-1.775214000	1.865025000
H	-5.100161000	-0.539551000	2.711162000
H	-6.421496000	-0.064740000	1.627996000
C	2.849628000	-2.678202000	-0.489314000
H	2.312089000	-3.061608000	0.378113000
H	2.584545000	-3.212607000	-1.400378000
C	3.810164000	-1.288971000	1.871108000
H	2.988031000	-1.949108000	2.120109000
H	4.148156000	-0.637111000	2.672473000
C	4.637599000	-1.555683000	0.789508000
H	5.612115000	-1.073444000	0.739354000
C	4.191917000	-2.266288000	-0.334157000
H	4.844543000	-2.302843000	-1.204558000

TS2

N	-2.701732000	1.306231000	-0.533652000
C	-2.893296000	1.541222000	0.848154000
C	-3.576437000	2.743786000	1.217113000
C	-3.998110000	3.634202000	0.268108000
C	-3.770658000	3.361003000	-1.113243000
C	-3.140132000	2.208528000	-1.483633000
C	-2.357724000	0.573007000	1.688249000
C	-2.436040000	0.613697000	3.192145000
C	-2.121448000	0.059345000	-0.812012000
C	-1.381731000	-0.185288000	-2.058053000
O	-1.370175000	0.523757000	-3.054362000
P	-1.672101000	-0.742032000	0.733456000
C	-3.003575000	-2.465031000	0.901946000
C	-3.033367000	-3.134074000	-0.347458000
Al	0.680194000	-1.530917000	1.050106000
Cl	0.587297000	-3.587693000	0.429757000
O	1.668613000	-0.515358000	0.082570000
C	2.562388000	0.557544000	0.353991000
C	3.731930000	0.531195000	-0.655185000
C	4.647582000	1.750545000	-0.417170000
C	3.880936000	3.079789000	-0.488266000
C	2.710841000	3.116325000	0.507949000
C	1.804109000	1.892889000	0.289521000
C	4.479441000	-0.830635000	-0.682209000
C	5.372382000	-0.967907000	-1.927807000
C	1.919862000	4.427977000	0.419789000
Cl	0.897637000	-1.328097000	3.187294000
C	5.289462000	-1.132557000	0.591169000
O	-0.707132000	-1.347596000	-1.955838000
C	0.127015000	-1.720352000	-3.069472000
C	-4.039847000	-1.243103000	-1.517254000
C	-3.550487000	-2.539536000	-1.505793000
H	-3.742923000	2.927091000	2.272872000
H	-4.509215000	4.546272000	0.562614000
H	-4.091894000	4.054164000	-1.882673000
H	-2.919693000	1.938735000	-2.506708000
H	-1.703356000	-0.061460000	3.643094000

H	-3.429873000	0.326059000	3.566832000
H	-2.220688000	1.619138000	3.574985000
H	0.899023000	-0.963061000	-3.221972000
H	-0.475193000	-1.826215000	-3.975841000
H	0.574010000	-2.670362000	-2.778898000
H	1.320227000	1.965462000	-0.696591000
H	1.000491000	1.882641000	1.039073000
H	1.472090000	4.549487000	-0.575872000
H	1.106797000	4.454796000	1.156549000
H	2.566720000	5.295656000	0.602884000
H	3.135379000	3.045516000	1.523018000
H	2.970913000	0.444838000	1.371311000
H	3.488415000	3.223790000	-1.507401000
H	4.564454000	3.918581000	-0.295064000
H	5.122230000	1.669046000	0.571353000
H	5.462957000	1.755930000	-1.151164000
H	6.146197000	-0.455278000	0.700610000
H	4.680627000	-1.053487000	1.499325000
H	5.685799000	-2.154960000	0.553846000
H	5.791981000	-1.979852000	-1.993172000
H	6.216879000	-0.266991000	-1.909192000
H	4.804868000	-0.782835000	-2.849307000
H	3.696572000	-1.595135000	-0.761673000
H	3.270735000	0.651130000	-1.649949000
H	-2.580006000	-3.014760000	1.741140000
H	-3.877636000	-1.877338000	1.177750000
H	-2.447547000	-4.043208000	-0.453879000
H	-3.382371000	-3.042598000	-2.455513000
H	-4.301131000	-0.760323000	-2.455122000
H	-4.457633000	-0.785109000	-0.627218000

TS3

C	6.424331000	-0.366843000	0.509786000
C	6.195760000	0.800455000	-0.278275000
C	4.921503000	1.145058000	-0.624522000
N	3.845866000	0.385971000	-0.212267000
C	4.023144000	-0.778546000	0.569816000
C	5.367311000	-1.133590000	0.914796000
C	2.510247000	0.609855000	-0.589013000
C	2.023372000	1.968025000	-0.876748000
O	2.703678000	2.975337000	-0.991038000
C	2.853222000	-1.429169000	0.937611000
C	2.800508000	-2.689831000	1.758320000
P	1.477293000	-0.639033000	0.184367000
Al	-0.589966000	-0.007042000	1.433856000
O	-1.793479000	0.039525000	0.200996000
O	0.681707000	1.948041000	-1.039523000
C	0.037731000	3.223678000	-1.238655000
Cl	-0.087794000	1.835377000	2.408890000
Cl	-0.783849000	-1.664326000	2.803053000
H	7.017872000	1.426094000	-0.607302000
H	4.670960000	2.025029000	-1.199027000
H	7.438330000	-0.646582000	0.780918000
H	5.517785000	-2.029120000	1.507209000
H	1.805533000	-2.841720000	2.186119000
H	3.046094000	-3.582127000	1.161676000
H	3.506381000	-2.650846000	2.596926000
H	-1.016968000	2.988105000	-1.373595000
H	0.181469000	3.850094000	-0.355523000
H	0.447808000	3.721277000	-2.121055000

C	-3.150540000	-0.397335000	0.180919000
C	-3.246173000	-1.737838000	-0.563023000
H	-3.502368000	-0.557952000	1.213166000
C	-4.686105000	-2.267066000	-0.681530000
H	-2.822210000	-1.601528000	-1.570203000
H	-2.615503000	-2.473077000	-0.045395000
C	-5.583718000	-1.195687000	-1.322239000
C	-4.737610000	-3.595730000	-1.446801000
H	-5.062547000	-2.451264000	0.338056000
C	-5.494526000	0.143788000	-0.575925000
H	-5.278783000	-1.052994000	-2.371311000
H	-6.625816000	-1.544208000	-1.345304000
C	-4.047055000	0.671305000	-0.482887000
H	-6.132546000	0.885447000	-1.072816000
H	-5.904493000	0.013765000	0.436104000
H	-3.665275000	0.782122000	-1.512037000
C	-3.924442000	2.069208000	0.183010000
H	-5.763297000	-3.981761000	-1.505568000
H	-4.368885000	-3.471948000	-2.474346000
H	-4.119375000	-4.360834000	-0.960216000
H	-2.848414000	2.253168000	0.290700000
C	-4.488605000	3.182537000	-0.717065000
C	-4.543070000	2.158300000	1.589477000
H	-4.300055000	3.124074000	2.049004000
H	-5.636945000	2.076423000	1.559478000
H	-4.166015000	1.377669000	2.260426000
H	-4.286351000	4.170704000	-0.284525000
H	-4.037601000	3.157038000	-1.718107000
H	-5.575915000	3.099741000	-0.841226000
C	0.679515000	-1.801414000	-1.487779000
H	0.075730000	-0.964006000	-1.838309000
H	0.071068000	-2.562625000	-1.002144000
C	2.403629000	0.021097000	-2.943184000
H	1.398951000	0.420024000	-2.868705000
H	3.148308000	0.696661000	-3.355680000
C	2.628605000	-1.348173000	-2.932110000
H	3.573545000	-1.730984000	-3.313359000
C	1.769082000	-2.239222000	-2.275158000
H	2.084632000	-3.276884000	-2.183231000

8a

C	-4.193116000	3.351718000	0.300067000
C	-4.278908000	2.973133000	-1.075952000
C	-3.684484000	1.807297000	-1.463233000
N	-3.036452000	1.001706000	-0.568307000
C	-2.911286000	1.330273000	0.793215000
C	-3.533930000	2.564730000	1.200499000
C	-2.390933000	-0.258886000	-0.976513000
C	-1.271296000	0.027824000	-1.999578000
O	-1.025246000	1.117340000	-2.467711000
C	-2.210032000	0.453370000	1.591320000
C	-1.969224000	0.650992000	3.067659000
P	-1.665754000	-0.952803000	0.649161000
Al	0.715337000	-1.644460000	0.713782000
O	1.618394000	-0.422180000	-0.094503000
O	-0.645584000	-1.109322000	-2.315239000
C	0.477472000	-1.010354000	-3.224571000
Cl	0.690552000	-3.572684000	-0.249439000
Cl	1.036849000	-1.807530000	2.844911000
H	-4.782858000	3.592993000	-1.807624000

H	-3.684464000	1.469898000	-2.491518000
H	-4.658348000	4.276533000	0.631247000
H	-3.467780000	2.843871000	2.245923000
H	-1.191115000	-0.023359000	3.437059000
H	-1.631507000	1.672307000	3.288882000
H	-2.873762000	0.467463000	3.667469000
H	0.831426000	-2.033764000	-3.340169000
H	0.146007000	-0.592583000	-4.178263000
H	1.248012000	-0.384441000	-2.773038000
C	2.543269000	0.565467000	0.356481000
C	1.852894000	1.937954000	0.371411000
H	2.855719000	0.327541000	1.385823000
C	2.791466000	3.084835000	0.784813000
H	1.451033000	2.134143000	-0.633904000
H	0.990726000	1.892795000	1.051046000
C	4.038513000	3.084891000	-0.113560000
C	2.069034000	4.438215000	0.767394000
H	3.126762000	2.894051000	1.817847000
C	4.737792000	1.717472000	-0.115129000
H	3.740694000	3.347364000	-1.141167000
H	4.739336000	3.864448000	0.217078000
C	3.794383000	0.575693000	-0.550260000
H	5.613312000	1.752392000	-0.775389000
H	5.121213000	1.515251000	0.895587000
H	3.428811000	0.817642000	-1.562799000
C	4.485178000	-0.812220000	-0.654786000
H	2.736723000	5.249359000	1.085442000
H	1.709092000	4.677484000	-0.242307000
H	1.200276000	4.436256000	1.437982000
H	3.685226000	-1.526108000	-0.888547000
C	5.488779000	-0.865557000	-1.820078000
C	5.154473000	-1.285290000	0.647900000
H	5.519189000	-2.313639000	0.533239000
H	6.017696000	-0.661663000	0.913457000
H	4.462209000	-1.279620000	1.497646000
H	5.866878000	-1.886524000	-1.957412000
H	5.024127000	-0.552843000	-2.764525000
H	6.357680000	-0.218331000	-1.645217000
C	-2.766222000	-2.440372000	0.980428000
H	-2.314966000	-3.275941000	0.427038000
H	-2.663659000	-2.677989000	2.044777000
C	-3.424020000	-1.266284000	-1.587534000
H	-2.855322000	-2.140883000	-1.923804000
H	-3.861008000	-0.817923000	-2.486678000
C	-4.501587000	-1.667224000	-0.610337000
H	-5.538352000	-1.490534000	-0.885398000
C	-4.193909000	-2.201806000	0.578793000
H	-4.979078000	-2.455880000	1.287686000

8a′

Al	0.599319000	-1.604377000	0.967303000
O	1.496761000	-0.471832000	0.030562000
O	-0.722439000	-1.343682000	-2.160310000
C	-1.219056000	-0.126259000	-1.936855000
H	3.037753000	2.963306000	1.687479000
N	-2.868660000	1.148718000	-0.607013000
C	-2.818287000	1.514505000	0.750987000
C	-2.266746000	0.606399000	1.628523000
P	-1.766362000	-0.870528000	0.770057000
C	-2.374411000	-0.204713000	-0.916805000

C	-3.547490000	-1.094463000	-1.482755000
C	2.417750000	0.564539000	0.365709000
C	3.588122000	0.578500000	-0.642681000
C	4.523958000	1.765923000	-0.329983000
C	3.780102000	3.109501000	-0.319502000
C	2.612357000	3.104589000	0.679989000
C	1.683306000	1.913787000	0.386623000
C	-3.349139000	2.815780000	1.070520000
C	-3.863698000	3.625189000	0.098485000
C	-3.890223000	3.201142000	-1.266489000
C	-3.384924000	1.970798000	-1.572170000
C	4.316381000	-0.790015000	-0.750535000
C	5.206926000	-0.866450000	-2.003427000
C	1.845087000	4.433092000	0.676348000
Cl	0.904847000	-1.524173000	3.104389000
Cl	0.602890000	-3.627146000	0.235441000
O	-0.850965000	0.891732000	-2.481636000
C	0.417369000	-1.431296000	-3.049035000
C	-2.107845000	0.841792000	3.110832000
C	5.125455000	-1.174809000	0.500956000
H	-4.283697000	3.834602000	-2.052298000
H	-3.349518000	1.591750000	-2.584788000
H	-4.259922000	4.601777000	0.364216000
H	-3.333746000	3.127161000	2.108759000
H	-1.397491000	0.135801000	3.550354000
H	-1.720820000	1.847626000	3.321152000
H	-3.057792000	0.735019000	3.656273000
H	0.641769000	-2.495260000	-3.109447000
H	0.159481000	-1.021746000	-4.028675000
H	1.251912000	-0.884934000	-2.607792000
H	2.824327000	0.378734000	1.372808000
H	1.196626000	2.055492000	-0.589666000
H	0.881621000	1.867785000	1.136697000
H	3.388772000	3.321706000	-1.327042000
H	4.478431000	3.922950000	-0.076965000
H	5.341092000	1.802830000	-1.061219000
H	4.994963000	1.616138000	0.652362000
H	3.132175000	0.768755000	-1.629056000
H	2.508221000	5.275953000	0.910585000
H	1.395927000	4.623590000	-0.307617000
H	1.035013000	4.428080000	1.416810000
H	3.524982000	-1.540518000	-0.870968000
H	5.509515000	-2.197844000	0.403609000
H	5.989741000	-0.514118000	0.644721000
H	4.520366000	-1.140532000	1.414232000
H	5.610520000	-1.878998000	-2.130248000
H	4.643933000	-0.615895000	-2.912240000
H	6.062039000	-0.181275000	-1.941740000
C	-3.327868000	-2.579267000	-1.354174000
C	-3.069372000	-3.136738000	-0.163826000
C	-2.970422000	-2.291221000	1.074286000
H	-2.858094000	-4.199275000	-0.082630000
H	-3.355684000	-3.184990000	-2.255473000
H	-4.442052000	-0.793583000	-0.920717000
H	-3.727039000	-0.817645000	-2.527324000
H	-2.608727000	-2.860524000	1.935418000
H	-3.932076000	-1.837269000	1.347558000

10

C	2.194282000	-0.396950000	-2.468349000

C	2.389145000	0.252233000	-1.054380000
P	1.467664000	-0.762179000	0.275797000
C	0.998747000	-2.321025000	-0.668595000
C	2.047620000	-2.736954000	-1.660195000
C	2.593993000	-1.851262000	-2.503404000
N	3.798103000	0.206161000	-0.622710000
C	4.799780000	0.802143000	-1.339142000
C	6.097856000	0.765206000	-0.919348000
C	6.393611000	0.101831000	0.312053000
C	5.401609000	-0.487132000	1.042370000
C	4.033084000	-0.463500000	0.591842000
C	2.944706000	-1.017754000	1.228346000
C	3.009382000	-1.734357000	2.554927000
C	1.900754000	1.715423000	-1.107169000
O	0.580315000	1.750043000	-1.310752000
C	-0.028215000	3.060752000	-1.404013000
Al	-0.571000000	0.105473000	1.371693000
Cl	-0.854815000	-1.395878000	2.907423000
O	2.621491000	2.685520000	-1.031581000
O	-1.794737000	0.085259000	0.156268000
C	-3.145151000	-0.365774000	0.156884000
C	-3.226050000	-1.738298000	-0.529078000
C	-4.660096000	-2.286729000	-0.628325000
C	-5.566058000	-1.253607000	-1.318193000
C	-5.494706000	0.116740000	-0.628022000
C	-4.053558000	0.663700000	-0.552250000
C	-4.696967000	-3.648789000	-1.333358000
C	-3.949069000	2.088347000	0.058216000
C	-4.573398000	2.225274000	1.458201000
Cl	0.004381000	2.018169000	2.168213000
C	-4.522278000	3.159200000	-0.886539000
H	6.869647000	1.248811000	-1.505900000
H	4.497476000	1.313125000	-2.243381000
H	7.419676000	0.066672000	0.668874000
H	5.617696000	-0.995159000	1.975320000
H	2.008540000	-1.922417000	2.953717000
H	3.518945000	-2.706829000	2.479354000
H	3.542427000	-1.140469000	3.309623000
H	-1.087023000	2.865486000	-1.563839000
H	0.129680000	3.603264000	-0.470465000
H	0.406512000	3.610992000	-2.242150000
H	-3.495270000	-0.485922000	1.195072000
H	-2.800467000	-1.640453000	-1.540452000
H	-2.589292000	-2.444080000	0.021280000
H	-5.037823000	-2.428264000	0.397542000
H	-5.257642000	-1.152063000	-2.371045000
H	-6.604272000	-1.614138000	-1.330939000
H	-6.139114000	0.829884000	-1.157464000
H	-5.907413000	0.023380000	0.386869000
H	-3.670250000	0.737543000	-1.584127000
H	-5.718766000	-4.047008000	-1.376861000
H	-4.327129000	-3.567662000	-2.364757000
H	-4.072567000	-4.385257000	-0.811733000
H	-2.875391000	2.288048000	0.162211000
H	-4.340829000	3.210074000	1.881428000
H	-5.666371000	2.132411000	1.427833000
H	-4.191783000	1.473648000	2.159003000
H	-4.333326000	4.165847000	-0.492221000
H	-4.067382000	3.100065000	-1.884487000
H	-5.608072000	3.058788000	-1.011125000
H	0.039968000	-2.097001000	-1.157192000

```
H        0.797209000     -3.094370000      0.080049000
H        1.136632000     -0.273864000     -2.730718000
H        2.764709000      0.182632000     -3.202479000
H        3.359347000     -2.159071000     -3.211348000
H        2.369723000     -3.776078000     -1.667654000
```

Table S2: Total energies of reactants, cycloadducts and transition structures in the gas phase and in methylene chloride**.**

	**Gas-phase**				**CH_2Cl_2**		
	**E [a] (a.u.)**	**ZPE[a] (a.u.)**	**E_{total} [b] (a.u.)**	**Relative energy (kcal/mol)**	**E [c] (a.u.)**	**E_{total} [d] (a.u.)**	**Relative energy (kcal/mol)**
**7a**	-2564.473593	0.467307	-2564.006286		-2564.487286	-2564.019979	
**9**	-156.001088	0.085242	-155.915846		-156.004287	-155.919045	
**TS1**	-2720.440134	0.554126	-2719.886008	22.67	-2720.455774	-2719.901648	23.45
**TS2**	-2720.441142	0.554551	-2719.886591	22.30	-2720.456779	-2720.902228	23.09
**TS3**	-2720.438421	0.554307	-2719.884114	23.86	-2720.454335	-2719.900028	24.47
**8a**	-2720.485900	0.557874	-2719.928026	-3.70	-2720.502533	-2719.944659	-3.54
**8a'**	-2720.482467	0.557735	-2719.924732	-1.63	-2720.499328	-2719.941593	-1.61
**10**	-2720.483562	0.557866	-2719.925696	-2.24	-2720.500623	-2719.942757	-2.34

[a] Energies calculated at the 6-31+G* level in the gas phase
[b] Energies of optimized molecule at the 6-31+G* level + ZPE at the same level
[c] Single-point energies of gas–phase-optimized geometries in CH_2Cl_2 at the 6-31+G* level
[d] Single-point energies in CH_2Cl_2 at the 6-31+G* level + ZPE in the gas phase at the same level
